# Histamine Induces Vascular Hyperpermeability by Increasing Blood Flow and Endothelial Barrier Disruption *In Vivo*

**DOI:** 10.1371/journal.pone.0132367

**Published:** 2015-07-09

**Authors:** Kohei Ashina, Yoshiki Tsubosaka, Tatsuro Nakamura, Keisuke Omori, Koji Kobayashi, Masatoshi Hori, Hiroshi Ozaki, Takahisa Murata

**Affiliations:** 1 Department of Veterinary Pharmacology, Graduate School of Agriculture and Life Sciences, The University of Tokyo, Tokyo 113–8657, Japan; 2 Department of Animal Radiology, Graduate School of Agriculture and Life Sciences, The University of Tokyo, Tokyo 113–8657, Japan; University of Illinois at Chicago, UNITED STATES

## Abstract

Histamine is a mediator of allergic inflammation released mainly from mast cells. Although histamine strongly increases vascular permeability, its precise mechanism under *in vivo* situation remains unknown. We here attempted to reveal how histamine induces vascular hyperpermeability focusing on the key regulators of vascular permeability, blood flow and endothelial barrier. Degranulation of mast cells by antigen-stimulation or histamine treatment induced vascular hyperpermeability and tissue swelling in mouse ears. These were abolished by histamine H1 receptor antagonism. Intravital imaging showed that histamine dilated vasculature, increased blood flow, while it induced hyperpermeability in venula. Whole-mount staining showed that histamine disrupted endothelial barrier formation of venula indicated by changes in vascular endothelial cadherin (VE-cadherin) localization at endothelial cell junction. Inhibition of nitric oxide synthesis (NOS) by L-NAME or vasoconstriction by phenylephrine strongly inhibited the histamine-induced blood flow increase and hyperpermeability without changing the VE-cadherin localization. *In vitro*, measurements of trans-endothelial electrical resistance of human dermal microvascular endothelial cells (HDMECs) showed that histamine disrupted endothelial barrier. Inhibition of protein kinase C (PKC) or Rho-associated protein kinase (ROCK), NOS attenuated the histamine-induced barrier disruption. These observations suggested that histamine increases vascular permeability mainly by nitric oxide (NO)-dependent vascular dilation and subsequent blood flow increase and maybe partially by PKC/ROCK/NO-dependent endothelial barrier disruption.

## Introduction

In allergic inflammation, antigen-stimulated mast cells release a range of mediators that can be subdivided into the preformed mediators, the synthesized lipid mediators, and the cytokines and chemokines. Histamine is a major preformed mediator released by mast cells and it strongly increases vascular permeability. This increase in vascular permeability is responsible for several features of acute allergic reactions including edema, urticaria, and anaphylactic shock in serious cases. Thus, the inhibition of vascular hyperpermeability has been recognized as an important therapeutic strategy for the treatment of allergic diseases.

The vasculature is mainly composed of vascular endothelial cells and vascular mural cells, such as vascular smooth muscle cells and pericytes. Endothelial cells form a monolayer covering the inner surface of the blood vessel, and mural cells cover the outside of this endothelial layer. The vascular structure varies depending on the type and/or site of the blood vessel. In the aorta, large veins, arteries, veins, and arterioles, endothelial cells are covered by at least one layer of vascular smooth muscle cells. In venules, the endothelial cells are covered by pericytes. Capillaries are composed solely of endothelial cells. Vascular mural cells and endothelial cells functionally interact and coordinate a variety of vascular functions.

Vascular permeability is determined by two major factors: blood flow and endothelial barrier function [1]. The contraction of vascular mural cells reduces downstream blood flow and limits vascular leakage. A clinical study showed that administration of a vasoconstrictor (phenylephrine) ameliorated rhinorrhea in patients with human allergic rhinitis [2]. In contrast, an animal study showed that vascular dilation by bradykinin evoked dye extravasation by increasing local blood flow in hamster cheek pouch vessels [3]. These results implied that mural cells play a crucial role in vascular permeability. However, there is a lack of evidence demonstrating the functional contribution of these cells to the modulation of vascular permeability.

The endothelial barrier is formed mainly by intercellular adherens junctions consisting of vascular endothelial cadherin (VE-cadherin), catenins, and the cytoskeleton [4]. Thrombin can increase endothelial permeability by activating calcium/RhoA-signaling, which disrupts endothelial adherens junctions [5]. Vascular endothelial growth factor (VEGF) also reduces barrier integrity by producing NO [6] or by activation of Src, which phosphorylates VE-cadherin. In contrast to these barrier-disrupting factors, Singleton et al. found that sphingosine-1-phosphate increased transendothelial electric resistance (TER) and enhanced the endothelial barrier via phosphoinositide 3 kinase/tiam1 Rac1 activation [7]. We also previously reported that prostaglandin D_2_ tightened adherens junctions and enhanced the endothelial barrier through a cAMP/protein kinase A (PKA)-dependent signal pathway [8]. Although many researchers have focused on endothelial barrier function, it is still unclear whether or how endothelial barrier formation modulates vascular permeability *in vivo*.

There have been several studies of the mechanisms underlying histamine-induced vascular permeability. Wessel et al. reported that histamine increased vascular permeability by phosphorylating VE-cadherin^Tyr685^ in murine cremaster tissue [9]. Moy et al. reported that histamine disrupted the barrier function of human umbilical vein endothelial cells (HUVECs) by increasing the intracellular calcium concentration [10]. Di Lorenzo et al. showed that Akt1-dependent NO production was crucial for histamine-induced endothelial barrier disruption [11]. These reports highlighted intracellular signaling pathways influencing histamine-induced changes in the endothelial barrier. However, the contribution of blood flow change to histamine-induced vascular hyperpermeability remains unknown.

In this study, we attempted to investigate the mechanism underlying histamine-induced vascular hyperpermeability *in vivo*, focusing on blood flow as well as endothelial barrier function. The present study revealed that histamine-induced hyperpermeability could mainly be attributed to the NO-induced blood flow increase, and partially to endothelial barrier disruption.

## Materials and Methods

### Ethics Statement

All animal experiments were approved by the institutional animal care and use ethical committees of the University of Tokyo (approval no. p11-578) and performed according to the National Institute of Health guidelines. General anesthesia was induced with 4% isoflurane via a nose cone and continued with 2% isoflurane during the procedures described. The anesthesia adequacy was monitored by the absence of eyelid reflex.

### Mice and *in vivo* treatments

Male and female FVB/NJcl mice (20–25 g) were purchased from CLEA Japan (Tokyo, Japan) and used for experiments. Mice transfected with enhanced green fluorescent protein (EGFP) with the chicken β-actin promoter were kindly provided by Prof. Masaru Okabe (Research Institute for Microbial Diseases, Osaka University) through the RIKEN BioResource Center (Tsukuba, Japan).

Histamine (0.4 mg in 20 μl 80% acetone) or 2-pyridylethylamine (1.2 mg in 60 μl 80% acetone) was applied transcutaneously to the ventral surface of the ear. Bradykinin (1 μg in 10 μl saline) or compound 48/80 (3 μg in 10 μl saline) was injected intracutaneously. Diphenhydramine (2.5 μg in 10 μl saline), cimetidine (2.5 μg in 10 μl saline), L-NAME (80 μg in 10 μl saline) or phenylephrine (1 μg in 10 μl saline) was injected intracutaneously 15 min before the reagent treatment. We confirmed that each solvent had no effect in each experiment.

### Reagents

The following reagents were obtained from the indicated suppliers: Evans blue, bradykinin, anti-dinitrophenol IgE and L-phenylephrine hydrochloride (Sigma-Aldrich, St. Louis, MO, USA); compound 48/80 (Nacalai tesque, Kyoto, Japan) formamide, histamine dihydrochloride, diphenhydramine, and cimetidine (Wako, Osaka, Japan); Y27632 (Roche Diagnostics Gmbh, Mannheim, Germany); 2-pyridylethylamine dihydrochloride (Tocris Bioscience, Bristol, UK); L-NAME (Enzo Life Sciences, New York, NY, USA); bisindolylmaleimide 1 and DNP-human serum albumin (Calbiochem, San Diego, CA, USA); endothelial growth medium-2 and Bulletkit medium (Lonza, Basel, Switzerland).

### Passive cutaneous anaphylaxis test

Passive cutaneous anaphylaxis (PCA) tests were performed as described previously [12], with slight modification. Briefly, an IgE-dependent reaction was generated by sensitizing the mouse ear with an intracutaneous injection of 30 ng anti- dinitrophenol (DNP) IgE in 100 μl of saline. Twenty-four hours after this sensitization, PCA was elicited by intravenous injection of 60 μg DNP-human serum albumin in 100 μl saline.

### Modified Miles assay

To assess vascular permeability, 50 mg/kg Evans blue was injected intravenously either concurrently with the DNP- human serum albumin challenge or 5 min after treatment. Thirty minutes after the injection, the ear thickness was measured using slide calipers. Then Mice were euthanized by cervical dislocation. The ear was dissected, dried in a constant-temperature oven, and weighed. Any extravasated Evans blue present in the ear was extracted in formamide, and quantified spectrophotometrically at a wavelength of 610 nm.

### Measurement of histamine

The ears were homogenized using an amalgam mixer (Retsch, Haan, Germany, mm300) and mixed with 150 μl of acetonitrile to precipitate protein. After centrifugation at 500 × *g* for 20 min, the supernatants were diluted to 10% acetonitrile with deionized water. The sample solution was then introduced into a liquid chromatography-tandem mass spectrometry 8030 system (Shimadzu, Kyoto, Japan) equipped with an electrospray (Turbospray) interface. The LC separation was carried out with a Kinetex C18 column (Phenomenex) using a mobile phase consisting of 0.02% (v/v) acetic acid (solvent A) and acetonitrile (solvent B). The following gradient was employed at a flow rate of 400 μl/min: A:B was initially 95:5; 5 min at 90:10; 6 min at 10:90. The LC-MS/MS was operated in the positive mode. For analysis, histamine was identified by the elution time (0.63 min) and by the ionic transition (m/z 119→95.2 and 119.0→41.25).

### *In vivo* microscopy

To visualize mouse ear vessels using a confocal microscope (ECLIPSE Ti with C1 confocal system, Nikon, Tokyo, Japan), 70 kDa fluorescein isothiocyanate-dextran (10 mg/kg in 100 μl phosphate-buffered saline, Sigma-Aldrich, St. Louis, MO, USA) was injected intravenously. Mice were then positioned on the microscope stage and their body temperatures were maintained at 37°C. Dextran leakage and vascular diameter were monitored every minute and quantified as described previously [13] using EZ-C1 FreeViewer (Nikon, Tokyo, Japan).

### Whole-mount and en face immunostaining

After treatment with each reagent, mice were euthanized by cervical dislocation and immediately perfusion-fixed with 4% paraformaldehyde. Their ears were then dissected and skinned. Samples were permeabilized with 0.15% Triton X-100 and incubated with blocking buffer, containing 5% normal donkey serum, for 30 min. Samples were probed with following primary antibodies overnight at 4°C: goat anti-VE-cadherin polyclonal antibody (Santa Cruz Biotechnology, Dallas, TX, USA), rat anti-CD31 monoclonal antibody (Biocare Medical, Concord, CA, USA), rabbit anti-desmin polyclonal antibody (Abcam, Cambridge, UK), or rabbit anti-FcεR1 polyclonal antibody (Upstate Biotechnology, New York, NY, USA). Samples were then probed with following secondary antibodies for 2 h at room temperature: Alexa Fluor 488 anti-goat IgG polyclonal antibody (Life Technologies, Carlsbad, CA, USA), Alexa Fluor 488 anti-rat IgG polyclonal antibody antibody (Life Technologies, Carlsbad, CA, USA), Alexa Fluor 594 anti-rabbit IgG polyclonal antibody antibody (Life Technologies, Carlsbad, CA, USA), and monoclonal anti-actin α-smooth muscle Cy3 (Sigma-Aldrich, St. Louis, MO, USA). Nuclei were labeled with DAPI (1 μg/ml) for 20 min prior to imaging using a confocal microscope. Since the diameter of artery is much smaller than that of vein, we can distinguish arteries and veins which run along together. Changes in VE-cadherin localization was quantified by measuring fluorescence intensity in three randomly selected areas using EZ-C1 Viewer (Nikon, Tokyo, Japan).

En face staining was performed as described previously [14]. Briefly, the external pulmonary artery was excised from mice, fixed with 4% paraformaldehyde, and permeabilized with 0.3% Triton X-100. The samples were then immunostained with the antibodies named above prior to imaging using a fluorescence microscope (Eclipse E800 Nikon, Tokyo, Japan).

### Blood flow velocity and volume measurements

Blood flow velocity (B_vel_) was calculated by measuring the velocity of EGFP-tagged blood cells in the mouse ear vessel. EGFP mouse plasma (20 μl) was collected and injected intravenously into FVB mice. After positioning the mice on the confocal microscope, each blood cell was monitored every 320 msec and the moving distance (MD) was measured. The average MD (MD_ave_) of 4 random blood cells was calculated. B_vel_ was then calculated according to the following formula: B_vel_ (μm/s) = MD_ave_ (μm) / 0.32 (s). In addition, blood flow volume (B_vol_) was calculated according to the formula: B_vol_ (μm^3^/s) = vessel diameter (μm)^2^ × 3.14 × B_vel_ (μm/s) / 4

### Laser doppler velocimeter measurements

After the mice had been anaesthetized, histamine was treated percutaneously. Changes in mouse ear blood flow were monitored with an Omegazone laser doppler blood-flow imaging system (Omegawave, Tokyo, Japan).

### Measurement of vascular contraction

The mouse mesenteric artery was excised and placed in physiological saline solution containing 136.9 mM NaCl, 5.4 mM KCl, 5.5 mM glucose, 23.8 mM NaHCO_3_, 1.5 mM CaCl_2_, 1.0 mM MgCl_2_, and 0.01 mM EDTA. After removing fat and connective tissue, the mesenteric arteries were cut into rings. In some experiments, the endothelium was removed by gently rubbing the intimal surface. Then the contractile force of the vascular rings was isometrically recorded with a force-displacement transducer (Orientec, Tokyo, Japan) connected to a strain amplifier (Yokogawa, Tokyo, Japan) under a resting tension of 10 mN. Precontraction was induced by phenylephrine (1 μM). Histamine (0.1–300 μM) was added cumulatively.

### Cell culture and TER measurement

Human dermal microvascular endothelial cells (HDMECs, Lonza, Basel, Switzerland) were cultured in Endothelial Growth Medium-2 containing 10% fetal bovine serum at 37°C in a humidified incubator with 5% CO_2_. Confluent cells (passage 4–8) were used after 4 h serum-starvation in Endothelial Basal Medium-2 with 2% bovine serum.

Endothelial barrier function was analyzed by measuring TER using the xCELLigence Real-Time Cell Analyzer DP system (Roche, Basel, Switzerland). This system monitored changes in TER over time across an interdigitated micro-electrode at the bottom of tissue culture E-plates (Roche, Basel, Switzerland). Cells (15000) were plated on E-plates and incubated until confluent. These cells were then stimulated by histamine (10 μM) and the TER was measured every 30 s. The TER was normalized to the value observed 1 h prior to stimulation.

### Statistical analyses

The results of the experiments were expressed as mean ± standard error of the mean (SEM). Statistical evaluation of the data was performed using an unpaired Student’s *t* test or by one-way analysis of variance (ANOVA) followed by Tukey's multiple comparison test. A value of *P* < 0.05 was considered to be statistically significant.

## Results

### Mast cell-induced vascular hyperpermeability was dependent on histamine

**Fig 1:**
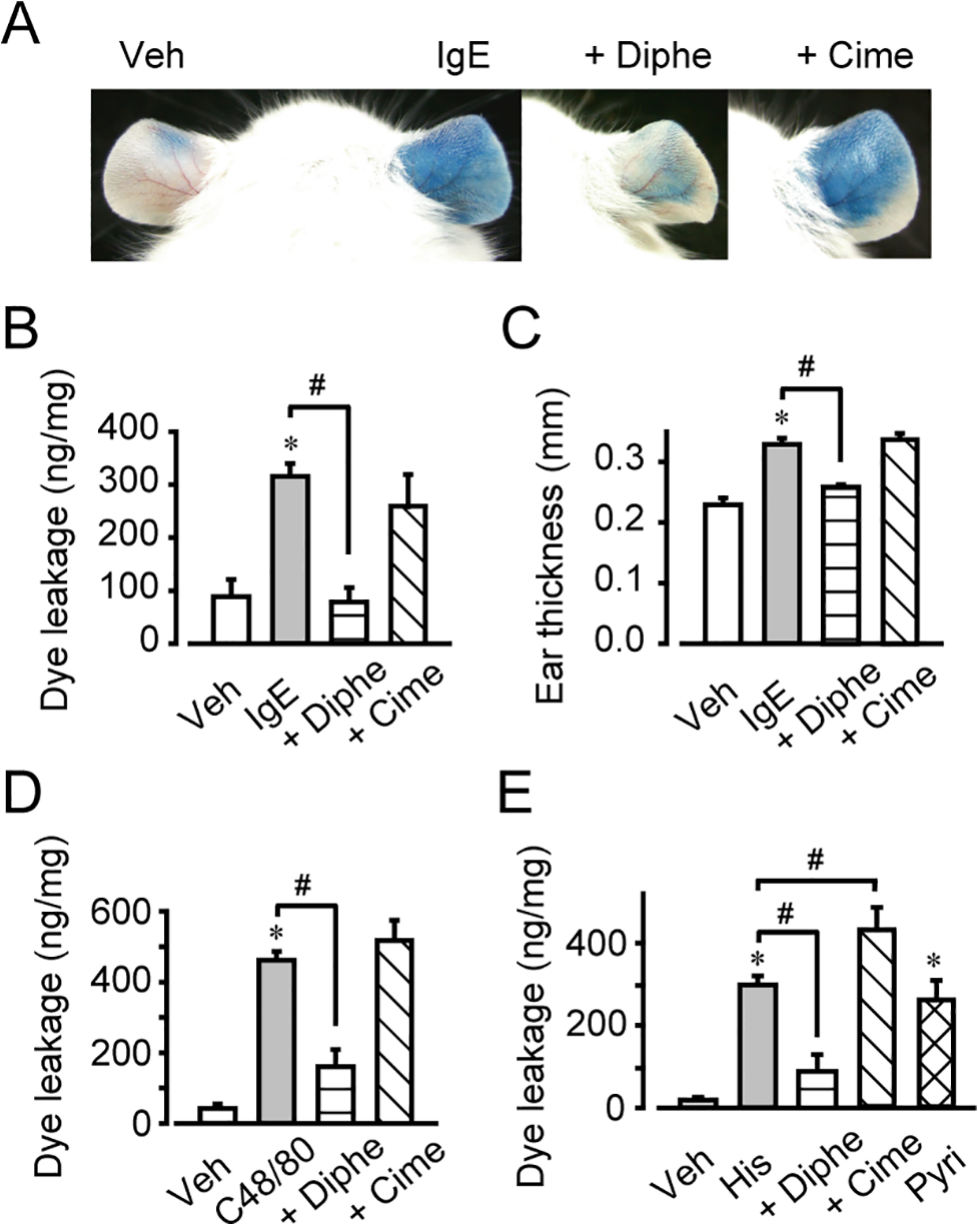
H1 receptor activation increased vascular permeability. The effects of diphenhydramine (Diphe) or cimetidine (Cime), 2-pyridylethylamine (Pyri) on vascular hyperpermeability. (A) Photographs of representative mouse ears. (B) Quantification of Evans blue leakage (n = 4–6). (C) Quantification of ear thickness (n = 4–6). #*P* < 0.05, as compared with IgE. (D) Quantification of Evans blue leakage after compound 48/80 (C48/80) application (n = 4–6). (E) Quantification of Evans blue leakage after histamine application (n = 4–7). *P < 0.05, compared with vehicle. #*P* < 0.05, compared with histamine. Data are presented as mean ± SEM.

Administration of antigen-specific IgE (30 ng/ear) and antigen (DNP, 60 μg/mouse) caused passive cutaneous anaphylaxis (PCA), with dye extravasation and ear tissue swelling (Fig 1A, 1B and 1C). Pretreatment with the histamine H1 receptor antagonist, diphenhydramine (2.5 μg/ear, 15 min), almost completely abolished these responses, while pretreatment with a H2 receptor antagonist, cimetidine (2.5 μg/ear, 15 min), did not (Fig 1A, 1B and 1C). Once histamine is released by mast cell degranulation, it is immediately metabolized by diamine oxidase in blood. PCA-inducing stimuli decreased the histamine content of the ear tissue, suggesting that histamine had been released from mast cells (S1 Fig). Intradermal injection of a mast cell activator, compound 48/80 (C48/80, 3 μg/ear), also immediately and strongly increased vascular permeability and caused tissue swelling (Fig 1D, S2 Fig). These responses were significantly inhibited by H1 blockade (diphenhydramine, 2.5 μg/ear) but not by H2 blockade (cimetidine, 2.5 μg/ear). These results suggested that mast cell degranulation induced vascular hyperpermeability through activation of H1 receptor-mediated signaling.

Exogenous administration of histamine (0.4 mg/ear) or an H1 agonist, 2-pyridylethylamine (1.2 mg/ear), increased vascular permeability and caused ear swelling (Fig 1E, S2 Fig). Consistent with the data described above, H1 blockade by diphenhydramine (2.5 μg/ear, 15-min pretreatment) abolished the histamine- induced inflammatory response. Interestingly, cimetidine (2.5 μg/ear, 15-min pretreatment) slightly but significantly enhanced the histamine-induced vascular hyperpermeability (Fig 1E, S2 Fig). H2 receptor signaling may therefore cause an anti-inflammatory effect by attenuating vascular leakage.

### Two types of vasculature are in mouse ear

**Fig 2:**
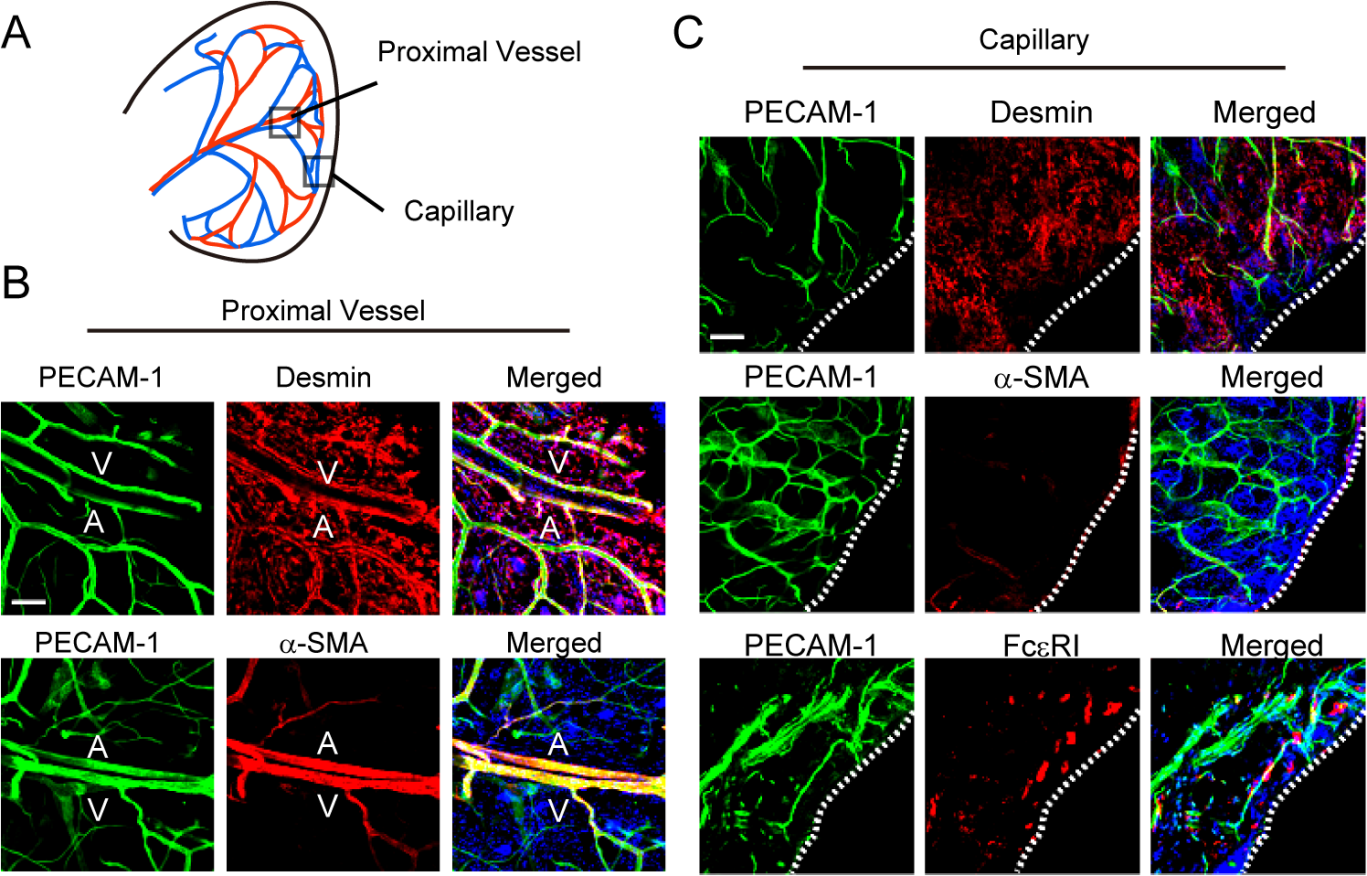
Two types of vasculature are in mouse ear. (A) Observation point. (B) Whole-mount immunostaining of PECAM-1, α-SMA, and desmin in the proximal vessel (magnification, ×200). Bar, 100 μm. A, artery; V, vein. (C) Whole-mount immunostaining of PECAM-1, α-SMA, desmin, and FCεRI in the capillary (magnification, ×200). Bar, 100 μm. Dotted lines indicate the edge of ear.

Whole-mount immunostaining revealed two types of vasculature in the mouse ear (Fig 2A). In proximal vessels, most platelet endothelial cell adhesion molecule-1 (PECAM-1)-positive endothelial cells were covered by mural cells, which were desmin-positive pericytes or α-smooth muscle actin (α-SMA)-positive smooth muscle cells (Fig 2B). In contrast, most capillaries were only composed of PECAM-1-positive endothelial cells (Fig 2A and 2C). FcεR1-positive mast cells also resided in the vicinity of the ear capillary (Fig 2C).

### Histamine increased vascular diameter and blood flow volume via H1 receptor activation

**Fig 3:**
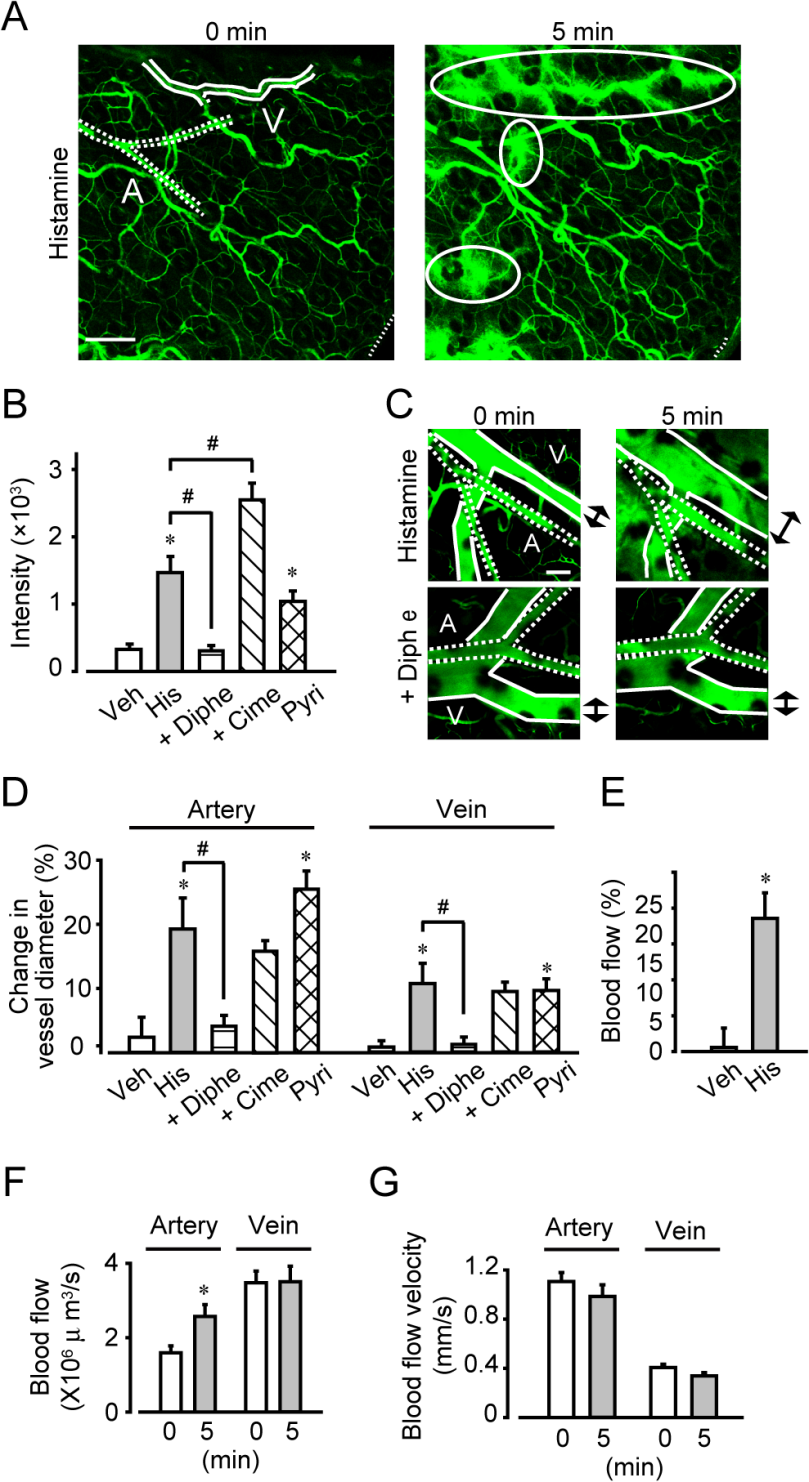
Histamine increased blood flow volume. (A) Typical images of the proximal vessel region before and 5 min after histamine treatment (magnification, ×100). Bar, 200 μm. Solid lines indicate vein. Dotted lines indicate artery. Ovoid circles indicate the FITC-dextran leakage. (B) Quantification of the FITC-dextran leakage after histamine application (n = 6–12). (C) Typical images of histamine-induced relaxation (magnification, ×200). Bar, 100 μm. A, artery; V, vein. (D) Quantification of the change in vessel diameter (n = 6–12). (E) Measurement of blood flow 5 min after histamine application using laser doppler velocimetry (n = 8). Measurement of blood flow (F) and blood flow velocity (G) before and 5 min after histamine application using *in vivo* microscopy (n = 15). *P < 0.05, compared with vehicle. #*P* < 0.05, compared with histamine. Data are presented as mean ± SEM.

We next observed the local vascular dynamics in the ear using *in vivo* imaging. As shown in Fig 3A, histamine (0.4 mg/ear) induced fluorescent dye leakage within 5 min after administration. Interestingly, histamine-induced dye extravasation was particularly marked around the venulae in distal vessels, but not in the arteries or capillaries (Fig 3A and 3B). Concomitantly with dye leakage, histamine rapidly dilated both the arteries and the veins, as evidenced by their increased diameters (Fig 3C, data obtained 5 min after histamine stimulation). Pretreatment with 2.5 μg/ear diphenhydramine (H1 antagonist) for 15 min completely inhibited both the vasodilation and the dye leakage, while the H2 receptor antagonist, cimetidine, (2.5 μg/ear, 15-min pretreatment) was not effective (Fig 3B, 3C and 3D). Consistent with these observations, H1 receptor stimulation (sole treatment with 0.4 mg/ear 2-pyridylethylamine) quickly dilated both arteries and veins (Fig 3D).

Vasodilation leads to an increase in local blood flow. Using laser doppler velocimetry, we confirmed that histamine (0.4 mg/ear) significantly increased the blood flow in mouse ear (Fig 3E). We next performed detailed observations using *in vivo* imaging. As shown in Fig 3F and 3G, histamine only increased blood flow in the artery (by approximately 1.5-fold, within 5 min) without changing the blood flow velocity (Fig 3F and 3G). Interestingly, these observations suggested that histamine increased blood flow in arteries, while causing hyperpermeability in veins.

### Histamine-induced NO-dependent vascular relaxation increased vascular permeability

**Fig 4:**
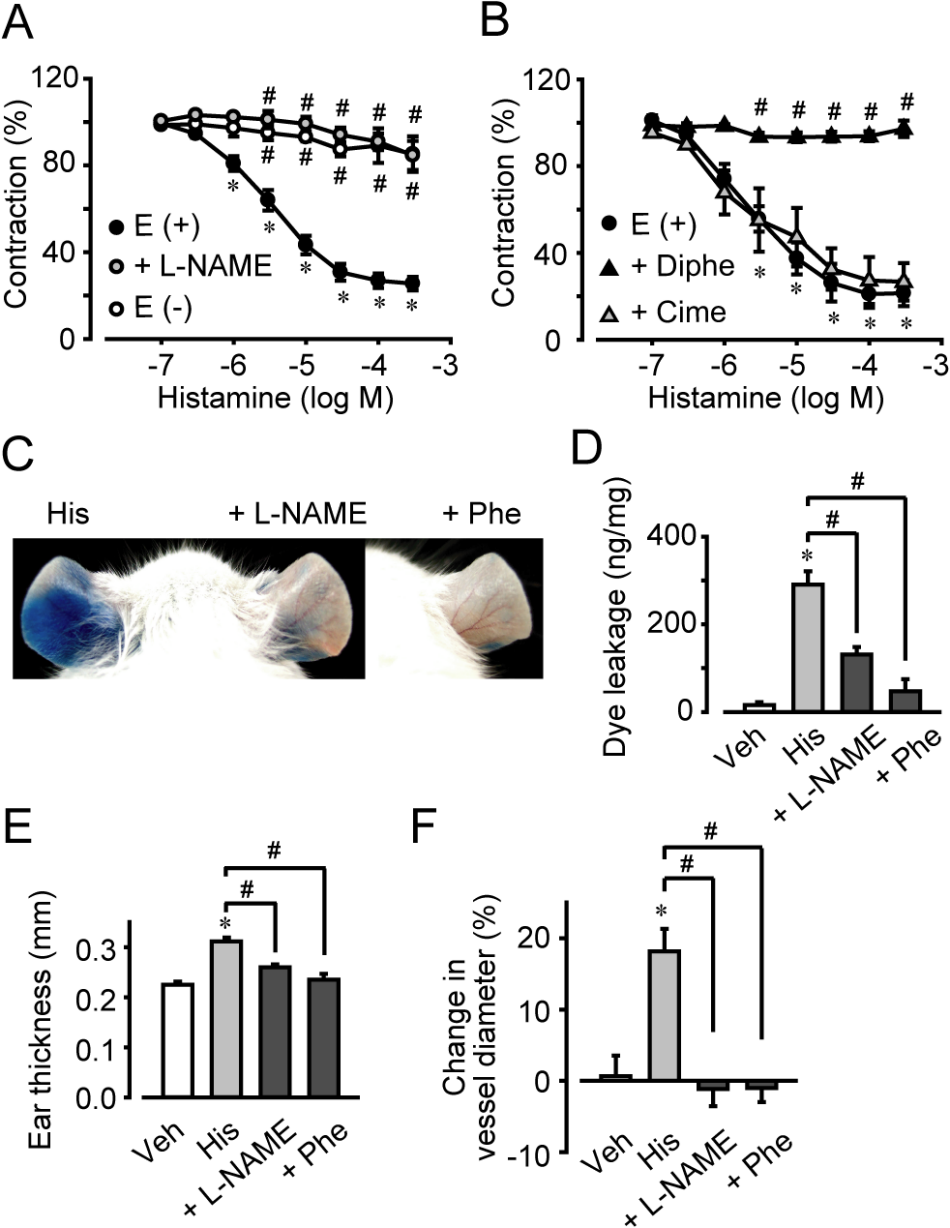
L-NAME or phenylephrine pretreatment inhibited histamine-induced hyperpermeability and vascular relaxation. (A) Effect of L-NAME pretreatment or endothelium on the histamine-induced relaxation of mesenteric artery. (B) Effect of diphenhydramine or cimetidine pretreatment on the histamine-induced relaxation of mesenteric artery(n = 4–5). (C) Effect of L-NAME or phenylephrine (Phe) on histamine-induced vascular hyperpermeability. Typical photographs showing extravasation of Evans blue after histamine treatment. (D) Quantification of the Evans blue leakage (n = 4–7). (E) Quantification of the ear thickness (n = 4–6). (F) Quantification of arterial diameter changes (n = 6–12). *P < 0.05, compared with vehicle. #*P* < 0.05, compared with histamine. Data are presented as mean ± SEM.

We next examined the effects of histamine on vascular relaxation and contraction using an isolated rat mesenteric artery preparation. As shown in Fig 4A, histamine (1–300 μM) induced dose-dependent relaxation of mesenteric artery in the presence of the endothelium (indicated as E (+)). Histamine (0.3 mM) decreased the contraction force to 26% of the precontraction elicited by 1 μM phenylephrine. Pretreatment with an NO synthase inhibitor, L-NG-nitroarginine methyl ester (L-NAME, 0.3 mM, 60 min), or removal of the endothelium (indicated as E (-)) completely inhibited histamine-induced vascular relaxation (Fig 4A). H1 antagonism by diphenhydramine (10 μM, 30-min pretreatment) also completely blocked the histamine-induced vascular relaxation, while H2 antagonism by cimetidine (10 μM, 30-min pretreatment) did not influence it (Fig 4B). These results suggested that activation of H1 receptor signaling induced endothelium-derived NO-dependent vascular relaxation.

We next examined whether the histamine-induced increase in blood flow caused hyperpermeability. As shown in Fig 4C, 4D and 4E, inhibition of NO synthase by L-NAME (80 μg/ear, 15-min pretreatment) decreased the histamine-induced (0.4 mg/ear) dye leakage to 63% and attenuated the tissue swelling to 59% of that observed in non-treated ears. Administration of a vasoconstrictor, phenylephrine (1 μg/ear, 15-min pretreatment), almost completely inhibited both the histamine-induced (0.4 mg/ear) dye leakage and the tissue swelling (Fig 4C, 4D and 4E). Similar observations were made in animals with mast cell degranulation-induced vascular leakage (S3 Fig). NO inhibition or vasoconstriction significantly inhibited the vascular hyperpermeability and tissue swelling induced by the mast cell activator, C48/80 (3 μg/ear). *In vivo* microscopy also revealed that NO-inhibition by L-NAME (80 μg/ear, 15-min pretreatment) or vascular contraction by phenylephrine (1 μg/ear, 15-min pretreatment) inhibited histamine-induced (0.4 mg/ear, 5 min) arterial dilation (Fig 4F). These results indicated that histamine-induced vascular hyperpermeability was attributable to NO-dependent vascular dilation and increased blood flow.

### Histamine disrupted the endothelial barrier *in vivo*

**Fig 5:**
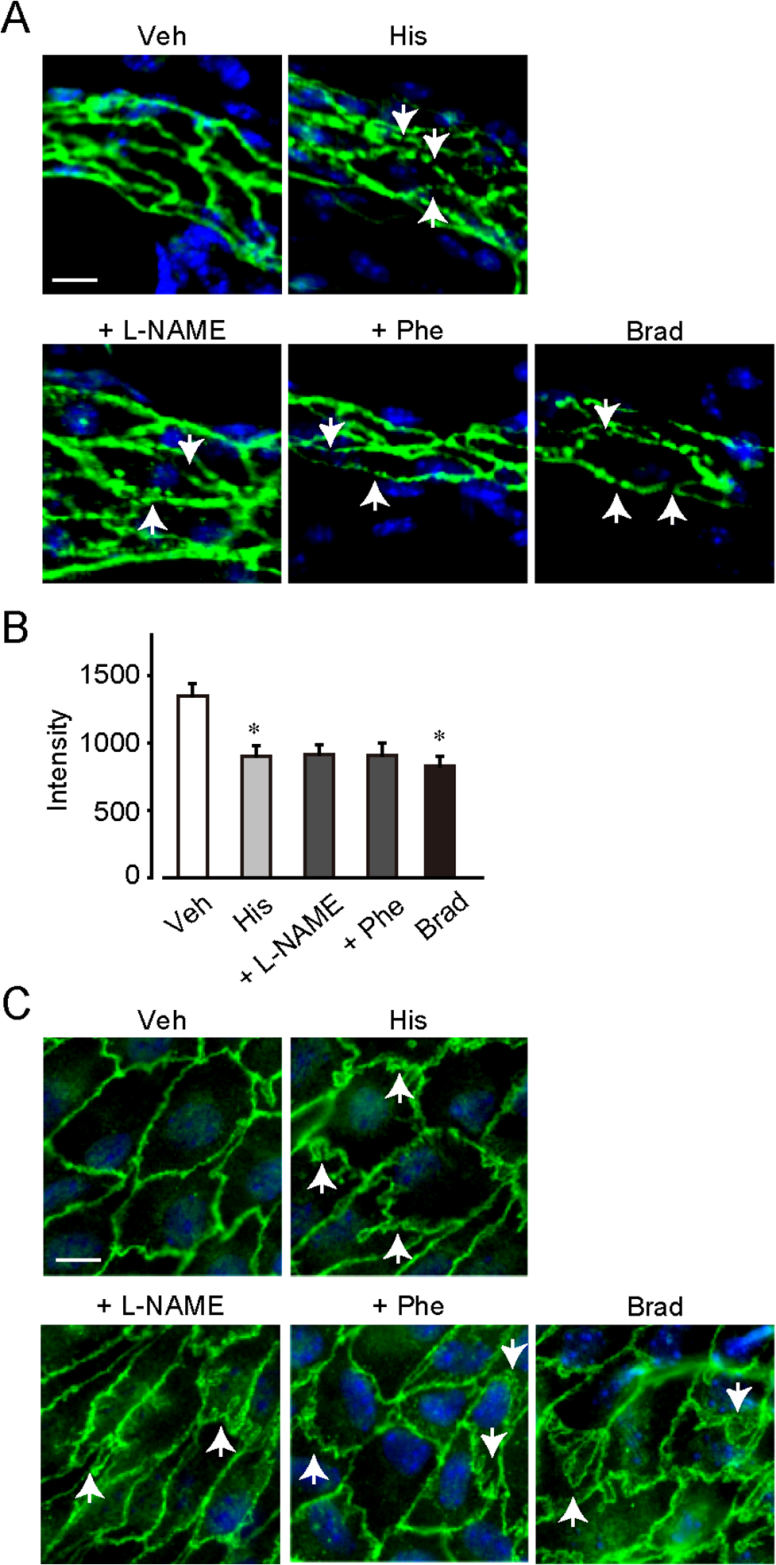
Histamine regulated endothelial barrier function *in vivo*. (A) Whole-mount immunostaining of VE cadherin in the ear vessel (magnification, ×400). Bar, 10 μm. (B) Fluorescence intensity of VE-cadherin at endothelial cell junction in ear vessel (n = 4–5). (C) En face immunostaining of VE-cadherin in the pulmonary artery (magnification, ×400). Bar, 10 μm. *P < 0.05, compared with vehicle. Data are presented as mean ± SEM.

We next assessed the effect of histamine on endothelial barrier formation by observing intercellular adherens junctions. Whole-mount immunostaining showed that an intracellular adhesion molecule, VE-cadherin, was located at cell-cell contact areas under non-stimulated conditions in venulae. Changes in VE-cadherin localization, internalization or disassembly, induce endothelial barrier disruption. Histamine (0.4 mg/ear, 10 min) caused partial internalization of VE-cadherin, as indicated by the arrows in Fig 5A (quantification of fluorescence intensity is shown in Fig 5B). NO inhibition by L-NAME (80 μg/ear, 15-min pretreatment) or vasoconstriction by phenylephrine (1 μg/ear, 15-min pretreatment) had no effect on this histamine-induced VE-cadherin internalization. Exposure to bradykinin (1 μg/ear, 10 min), a well-characterized disruptor of the endothelial barrier, also triggered VE-cadherin internalization.

Fig 5C shows that similar results were obtained by en face immunostaining of the pulmonary artery. VE-cadherin was located at cell-cell contact areas in the absence of stimulation and histamine (10 μM) disassembled VE-cadherin, as indicated by the arrows (Fig 5C). NO inhibition by L-NAME (3 mM, 60-min pretreatment) or vasoconstriction by phenylephrine (1 μM, 15-min pretreatment) did not influence the histamine-induced VE-cadherin disassembly. Again, treatment with bradykinin (0.1 μM) also caused VE-cadherin disassembly (Fig 5C).

These results suggested that histamine disrupted vascular endothelial barrier function through an NO-independent signaling pathway.

### Histamine regulated endothelial barrier function in vitro

**Fig 6:**
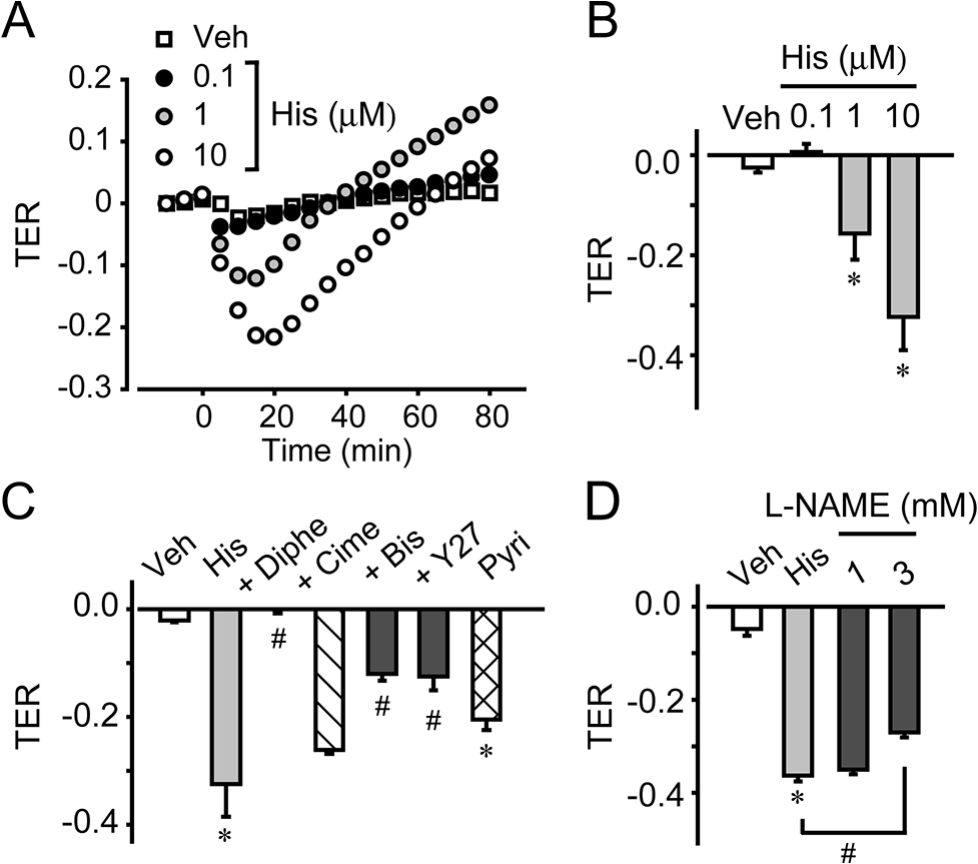
Histamine regulated endothelial barrier function *in vitro*. (A) Typical graph of histamine treatment in HDMECs. (B) Quantification of TER in HDMECs (n = 4–8). (C) Effects of pretreatment with diphenhydramine, cimetidine, Y27632, and bisindolylmaleimide 1 on the histamine-induced change in transendothelial electric resistance (TER) in HDMECs (n = 4–6). (D) Effect of pretreatment with L-NAME on the histamine-induced change in TER in HDMECs (n = 4). *P < 0.05, compared with non-treated control. #*P* < 0.05, compared with histamine. Data are presented as mean ± SEM.

Endothelial barrier function was evaluated *in vitro* assay by measuring TER. A low concentration of histamine (0.1 μM) slightly decreased the TER of HDMECs, but this effect was not statistically significant (Fig 6A and 6B). At higher concentrations, histamine (1–10 μM) decreased the TER of HDMECs in a dose-dependent manner, and these decreases lasted for about 40 or 60 min. H1 blockade by diphenhydramine (10 μM, 30-min pretreatment) almost completely inhibited the histamine (10 μM)-induced decrease of TER (Fig 6C) but H2 blockade by cimetidine (10 μM, 30-min pretreatment) did not have this effect. Treatment with the H1 agonist, 2-pyridylethylamine (10 μM), consistently decreased the TER (Fig 6C). Pretreatment with an inhibitor of Rho-associated, coiled-coil containing protein kinase (ROCK), Y27632 (10 μM, 60 min), or a protein kinase C (PKC) inhibitor, bisindolylmaleimide 1 (1 μM, 30 min), inhibited the histamine-induced decrease in TER (Fig 6C). Pretreatment with L-NAME (1 mM, 30 min) did not influence this effect, while pretreatment with 3 mM L-NAME for 60 min slightly inhibited the histamine-induced decrease in TER. These *in vitro* results suggested that histamine disrupted endothelial barrier function, mainly via H1/PKC/ROCK/NO signaling.

## Discussion

During allergic inflammation, activated mast cells release a large amount of histamine, leading to vascular hyperpermeability [15, 16]. Since vascular hyperpermeability is closely associated with various allergic symptoms such as urticaria, conjunctivitis, and nasal congestion, understanding the mechanism underlying histamine-induced vascular hyperpermeability will provide novel therapeutic insights into allergic diseases. Several groups have previously reported that histamine disrupted the endothelial barrier [11, 17, 18]. However, many of these studies focused on cell-based *in vitro* systems and the precise mechanism involved *in vivo* still remained unclear. The present study revealed that histamine-induced hyperpermeability could mainly be attributed to the NO-induced blood flow increase, and partially to endothelial barrier disruption. Vasoconstrictors are currently used to treat rhinitis [2]. Our observations supported the effectiveness of these treatments and implied that they may also have therapeutic efficacy for other allergic symptoms, including urticaria.

Previous studies suggested that increased blood flow elevated the hemodynamic forces on the vascular wall in the form of shear stress and intravascular hydrostatic pressure, thus compromising its barrier function. Orsenigo et al. showed that shear stress led to VE-cadherin phosphorylation and internalization, followed by adherens junction disassembly [19]. Other researchers indicated that an increase in intraluminal hydrostatic pressure intensified the outward transfer of plasma components [1]. The present study showed that histamine simultaneously increased blood flow, altered VE-cadherin localization, and caused vascular hyperpermeability. NOS-inhibition or vascular contraction were found to decrease blood flow, inhibiting vascular leakage without affecting VE-cadherin localization. We previously reported that an inflammatory mediator, prostaglandin E_2_, or the vasorelaxant, isoproterenol, elicited vascular hyperpermeability by increasing blood flow while causing VE-cadherin accumulation at intercellular boundaries, indicating enhancement of the endothelial barrier [13]. Although it is still unclear how increased hemodynamic force causes the extravasation of plasma components, these observations support the idea that vascular dilation and the subsequent increase in blood flow are major *in vivo* determinants of vascular hyperpermeability.

We revealed that histamine induced hyperpermeability of venulae, while only elevating blood flow in arteries. This result was consistent with previous *in vivo* studies showing that leukotriene mainly increased vascular permeability in venulae [20]. Compared with arteries and large veins, venulae have a thinner vessel wall and smooth muscle layer. The structural weakness of venulae may be responsible for their permeability. In addition, the venous endothelial layer is reported to be more permeable than that of arteries because it expresses fewer cell-cell adhesion proteins [21]. This is one potential explanation for our observation that histamine induced increased arterial blood flow and increased permeability of venulae.

Endothelial barrier function is also critical for vascular permeability. Mikelis et al. recently suggested the functional importance of endothelial H1 receptor-signaling in histamine-induced vascular leakage [22]. In our study, pretreatment with L-NAME or phenylephrine significantly decreased, but did not perfectly suppressed the histamine-induced vascular leakage without changing VE-cadherin mis-localization (Figs 4C, 4D, 4E, 5A, 5B and 5C). These results suggest that endothelial barrier disruption as well as blood flow increase is included in the histamine-induced vascular leakage.

Several inflammatory substances such as bradykinin are known to induce vascular hyperpermeability by disrupting this barrier in isolated endothelial cells [23]. Histamine is also known to increase endothelial permeability in HUVECs [10]. Consistent with these reports, we found that histamine disrupted adherence junction assembly *in vivo* and *in vitro*. PKC and ROCK are well-known signaling molecules involved in mediating the endothelial barrier. Activation of these kinases induces drastic cytoskeletal rearrangement, including actin stress fiber formation and myosin light chain phosphorylation. Both of these effects result in adherens junction disassembly and endothelial hyperpermeability in human pulmonary artery endothelial cells [24]. These findings indicated that PKC/ROCK activation and the subsequent cytoskeletal rearrangement mediated histamine-induced endothelial barrier disruption.

NO is another well-known regulator of endothelial barrier function. Several barrier-disrupting substances, including platelet-activating factor and VEGF, exert their actions through effects on endothelial NO production, causing adherens junction destabilization [6, 25, 26]. Di Lorenzo et al. revealed that the barrier-disrupting action of histamine was fully dependent on NO production in human dermal microvascular endothelial cells [11]. However, our *in vivo* observations showed that inhibition of NO did not restore histamine-induced changes in VE-cadherin localization, even though it completely blocked vascular dilation and leakage. *In vitro* experiments showed that L-NAME only slightly attenuated the histamine-induced endothelial barrier disruption, even at a high concentration (3 mM). Histamine-induced NO production may therefore only partially affect the properties of the endothelial barrier, whilst strongly inducing vasodilation. Further investigation is required to clarify this discrepancy.

In conclusion, the present study showed that *in vivo* histamine-induced hyperpermeability was dependent predominantly on NO-mediated dilation of vascular smooth muscle and the subsequent blood flow increase, and partially on PKC/ROCK/NO-dependent endothelial barrier disruption. Vascular mural cells and endothelial cells work together to control a range of vascular functions. Since the structure and cellular components of the vasculature vary by tissue type and site, comprehensive *in vivo* studies focusing on both functions are required to fully elucidate the pathophysiological implications of vascular permeability.

## Supporting Information

S1 Fig PCA reaction decreased histamine content in the ear. PCA reaction decreased histamine content in the ear. Histamine level in the ear (n = 5). *P < 0.05, compared with vehicle. Data are presented as mean ± SEM.
(TIF)

S2 Fig Endogenous and exogenous histamine induced ear swelling. Effect of diphenhydramine or cimetidine on C48/80-induced vascular hyperpermeability. (A) Typical photographs of mouse ears. (B) Quantification of the ear thickness (n = 4–6). #*P* < 0.05, compared with C48/80. Effect of diphenhydramine or cimetidine on histamine-induced vascular hyperpermeability. (C) Typical photographs. (D) Quantification of the ear thickness (n = 4–7). *P < 0.05, compared with vehicle. #*P* < 0.05, compared with histamine. Data are presented as mean ± SEM.
(TIF)

S3 Fig C48/80 induced vascular hyperpermeability by vascular relaxation. Effect of L-NAME or phenylephrine on C48/80 or histamine-induced vascular hyperpermeability. (A) Typical photographs of extravasation of Evans blue after C48/80 treatment. (B) Quantification of the Evans blue leakage after C48/80 treatment (n = 4). (C) Quantification of the ear thickness after C48/80 treatment (n = 4). #*P* < 0.05, compared with C48/80. *P < 0.05, compared with vehicle. #*P* < 0.05, compared with histamine. Data are presented as mean ± SEM.
(TIF)
