# Supplementary figures and images for: Histamine Induces Vascular Hyperpermeability by Increasing Blood Flow and Endothelial Barrier Disruption In Vivo
Source: PLoS One. 2015 Jul 9;10(7):e0132367. doi: 10.1371/journal.pone.0132367 (PMC4497677; doi:10.1371/journal.pone.0132367)

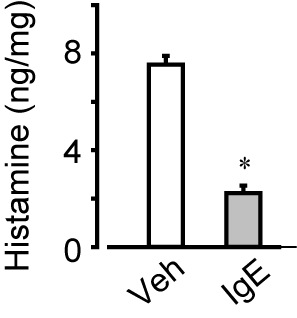

Supplement: S1 Fig — PCA reaction decreased histamine content in the ear. Histamine level in the ear (n = 5). *P < 0.05, compared with vehicle. Data are presented as mean ± SEM. (TIF) [file pone.0132367.s001.tif]

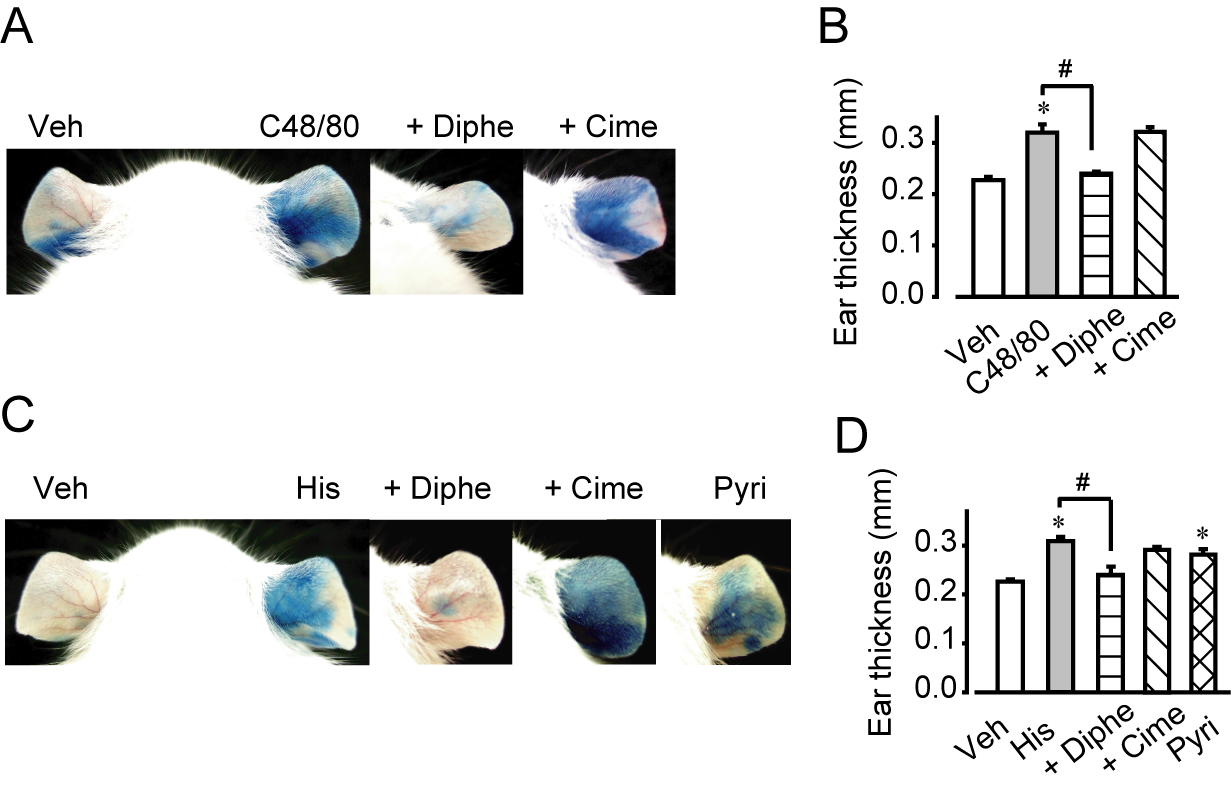

Supplement: S2 Fig — Effect of diphenhydramine or cimetidine on C48/80-induced vascular hyperpermeability. (A) Typical photographs of mouse ears. (B) Quantification of the ear thickness (n = 4–6). #P < 0.05, compared with C48/80. Effect of diphenhydramine or cimetidine on histamine-induced vascular hyperpermeability. (C) Typical photographs. (D) Quantification of the ear thickness (n = 4–7). *P < 0.05, compared with vehicle. #P < 0.05, compared with histamine. Data are presented as mean ± SEM. (TIF) [file pone.0132367.s002.tif]

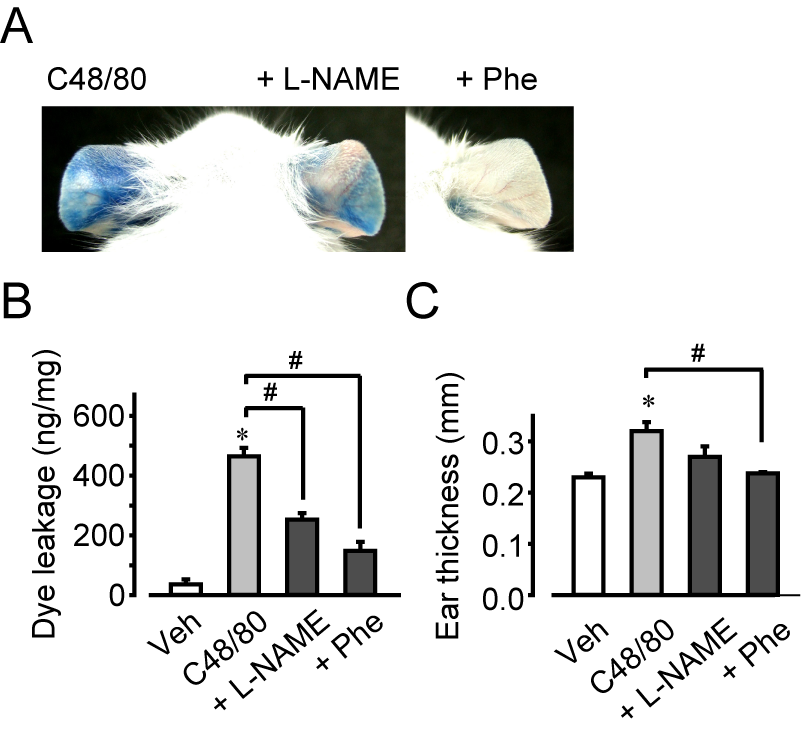

Supplement: S3 Fig — Effect of L-NAME or phenylephrine on C48/80 or histamine-induced vascular hyperpermeability. (A) Typical photographs of extravasation of Evans blue after C48/80 treatment. (B) Quantification of the Evans blue leakage after C48/80 treatment (n = 4). (C) Quantification of the ear thickness after C48/80 treatment (n = 4). #P < 0.05, compared with C48/80. *P < 0.05, compared with vehicle. #P < 0.05, compared with histamine. Data are presented as mean ± SEM. (TIF) [file pone.0132367.s003.tif]
